# The absence of changes in the relative age effect present an opportunity for lower income soccer clubs to be more efficient than Europe’s elite

**DOI:** 10.1371/journal.pone.0323971

**Published:** 2025-05-20

**Authors:** Thomas P. Craig, Kevin Enright, Patrick Maughan, Will Abbott, Javier Fernandez Navarro

**Affiliations:** 1 School of Pharmacy, Applied Sciences and Public Health, Robert Gordon University, Aberdeen, United Kingdom; 2 Research Institute for Sport and Exercise Sciences, Liverpool John Moores University, Liverpool, United Kingdom; 3 Charlton Athletic Football Club, London, United Kingdom; Portugal Football School, Portuguese Football Federation, PORTUGAL

## Abstract

An overrepresentation of athletes born earlier in the year compared with those born later in the year is known as the relative age effect (RAE). This is perceived to be due to physical selection bias which leads to higher degrees of exposure to coaching, physical training and competition at a younger age. Even with increasing knowledge and established interventions, clubs in Europe’s top leagues still present a strong RAE. Scottish clubs have limited resources in comparison meaning academy efficiency is paramount. The main study aim was to assess changes in the RAE over a ten-year period in Scottish soccer. A secondary aim was to establish if physical differences exist across each quarter due to findings in English academy players that maturation status and not RAE is the main discrepancy for physicality. A retrospective analysis of 512 players from a Scottish academy over a ten year period was granted ethical approval. The impact of relative age effect was assessed against anthropometric and physical characteristics. The range of players in each quarter was Q1 37.0–42.9% versus Q2 22.8–32.4%, Q3 11.9–26.0% and Q4 7.1–14.3% with no impact of time on RAE profiles. Odds Ratio analysis indicate a greater chance of selection within the academy when assessing Q1vsQ4 players quarter comparisons (ranging 3.2–5.2 times more likely to be signed). When controlling for age group, multilevel modelling showed there were no significant differences across quarters in physical measures with the exception of a trivial CMJ difference. The lack of progression in the RAE profiles is disappointing however presents an opportunity for increased efficiency. By viewing the RAE as an under representation of Q4 players and using established corrective procedures, this can contribute to the unnecessary release of players from academies due to RAE, thus addressing challenges in financially restricted environments that resource rich environments such as Europe’s elite have not yet overcome.

## Introduction

Youth soccer leagues chronologically match children based on their birth year with the aim of matching developmental milestones to ensure fair competition. An overrepresentation of athletes born earlier in the year compared with those born later in the year is known as the relative age effect (RAE) [1]. This may be in part due to a physical selection bias for example increased stature, muscle mass, sprint speed etc in older children [2]. The selected children enter talent development systems earlier relative to their age (e.g., two U11 players entering at the same time may contain a December born player who is 12 months younger than a January born player although both are beginning their academy journey during the same section period) [3] and gain higher degrees of exposure to coaching, physical training and competition at a younger age which in turn can lead to an improvement in technical and tactical abilities at older ages [4]. Although this primarily relates to physical advantages as older players are assessed against their younger peers [5–7] and leads to improvements in physical performance associated with the impact of stature and muscle mass [2,6] this also impacts sociological and psychological advantages [8]. The RAE expresses itself with the increasing competition level in youth soccer [8], and impacts coaches’ subjective measures of player ability, with these subjective measures of ability being the determining factor of inclusion and exclusion in a soccer academy [2]. The effects of RAE bias persist through to senior/adult level and could influence the judgement of decision makers explaining increased dropout of players who have not yet fulfilled their potential [2,4,9].

Clubs with significant financial resource do not need to consider the established requirement to attenuate the costs of recruiting and developing academy players [5]. Thus, interventions can be utilised to alleviate the RAE without considering financial impact. These include manipulation of chronological banded categories with additional squads [10] with significant cost implications which could be up to double that of standardised age bands, and free interventions such as players playing up or down an age category [4]. Given the resource available, it is thus surprising that the analysis of the FC Barcelona Sporting Model showed significant relative age effects throughout their soccer academy [8]. Odds ratios were utilised within the FC Barcelona Sporting Model [8] on a retrospective analysis which evidenced an under representation of players born in Q4 (Oct-Dec) in all age groups. Players born in Q1 (Jan – Mar) were 7.5–18.6 times more likely to be part of the academy versus Q4 players across all age groups cumulating in Q1 first team players being 3.3 times more likely to be part of the first team squad against their Q4 teammates. The academy RAE impact is visible across the birth year with Q2 (Apr – Jun) born players being 3.4–9.8 times and Q3 (Jul-Sep) born players being 1.6–3.7 times more likely to be part of the academy versus those born in Q4. Ultimately clubs who persist with a relative age effect show an inefficiency in their recruitment and transition processes, as they may not be capturing the players with the best potential when recruitment is influenced by age at early academy age groups.

RAE analysis across Europe’s elite leagues reflects the patterns observed at FC Barcelona [10]. A ten year analysis of professional players across UEFA’s top ten European leagues showed the existence of an RAE in professional soccer [10]. Collectively in the leagues, the RAE assessed as players born in Q1 vs Q4 increased in the ten years from 2000 (29.3 vs 19.8%, P < 0.05) to 2010 (31.9 vs 18.4%, P < 0.05) with the RAE becoming more pronounced with time. Despite solutions being proposed in the literature, this would indicate over that time period these solutions are not being utilised effectively within the top European leagues [10]. Although the leagues assessed by Helsen [10] were at first team professional level, these results may be due to the recruitment of first team players from already biased academy selection pools [4,11] and do not consider leagues with low player pool due to lower populations and often greater financial restrictions such as Scotland. The increase in foreign players evidenced by Helsen [10] (33.5–43.4%) across the same time period supports the narrative that the finance available in these top leagues means clubs can simply purchase players if their academy is not producing. Of the 100 most valuable players 60% were born in the first half of the year with the mean player value being $8.5m greater in the players born at the start of the year [12].

Previous analysis has however only looked at RAE and not integrated physical characteristics. More recent work has established a need to consider the physical impact of maturation status even when there is an established RAE [13]. With an age distribution across 426 English players that contained a strong RAE, Patel and colleagues [14] investigated the differences in physical performance and anthropometric characteristics across birth quarters Q1: 43.4%; Q2: 29.8%; Q3:19.5%; Q4: 7.3%. The distribution ranged from 27.3% and 61.3% in Q1 to 3.2% and 14.7% in Q4 for the individual age groups (360 youth and 66 first team registered players between 2010 and 2018). Both anthropometric and physical measures were assessed across each age group with only limited differences in selective measures throughout the analysis (no differences: Under 13s (U13), U15, U21). Surprisingly where differences did exist, players born in Q4 displayed better physical performance than players born in other quarters (YoYo Intermittent Recovery Test (YYIR1): U11, U12 U14; agility and sprint: U16 and U18). Only in the first team was there a negative impact of a Q4 birth (in counter movement jump only) and across all age groups there were no differences in anthropometric characterises. Patel et al.[14] also evidenced that Q4 players reached peak height velocity at a younger age and the authors concluded that relatively younger players demonstrating advanced growth and/or maturation have an enhanced likelihood of being selected into an academy regardless of their birth dates. Similar results observed in Spain were attributed to maturation status being the main contributor of physical performance [15] with relative age not impacting the physical performance measures assessed. Although there was a RAE present within a well funded English academy, there was also a distinct selection bias favouring players advanced in maturation from U12 level which increased with age [16]. Maturation and relative age should thus be considered as two separate constructs when assessing the overall impact within a talent ID and development environment [13].

Given the inefficiency of resource rich clubs [8,10], efficiency opportunities may be of significant interest to clubs with restrictions in regards to income streams and player pool. Whilst it has been suggested that future studies should focus on the reduction of the RAE as opposed to historical assessment of the RAE [17], it is still imperative that coaches, practitioners and recruiters understand patterns in their own players recruitment and transition occurring within their unique environments [18]. Our previous work [11] in Scottish soccer identified strong relative age bias within the entire academy cohort and players awarded professional contracts between 2006 and 2016 however an analysis of the impact of the increase in RAE knowledge over time [17] would be beneficial to establish if clubs are being more efficient in their talent ID and development strategies with time. Financial limitations exist in Scottish soccer which limit the feasibility of costly interventions (such as a move to 6 month age bands). With the lower player pool and financial resource compared with other European nations, clubs in Scotland must optimise their talent development and identification processes. It is firstly however important to assess the longitudinal impact and development of the academy [19] in this instance related to the impact of relative age and whether scope for further efficiency is available. The main study aim was therefore to assess any changes in the relative age effect over a ten year period in an elite Scottish soccer academy within the general academy player pool and those awarded professional contracts. A secondary aim was to establish if physical differences exist across each grouped quarter in order to assess the impact of any potential RAE on physical performance.

## Methodology

### Participants

A retrospective analysis of 512 male academy players (from 2005–06 to 2016–17) from the Scottish Academy of a professional club was completed. Players from U11 to U17s were assessed for physical performance characteristics and relative age effect impact. The academy held “elite” categorisation from the Scottish FA’s Club Academy Scotland criteria.

The retrospective analysis was granted ethical approval by the review board at Robert Gordon University (Ethics approval ID SHS/19/44). Players (or parents/ guardian where appropriate) provided informed consent via the contractual process and the data analysed was originally collected for sports science testing and delivery purposes [20]. Author’s TC and KE had previous access to the data due to practitioner roles within the club involving data collection. All other author’s gained access to anonymised data following ethical approval with this being shared from 3rd May 2024 for discussion. Following discussion only TC and JFN retained access with anonymised copies of the raw data being retained in accordance with their respective institution’s data security procedures.

Players were repeatedly tested throughout their time at the academy between 2 and 3 times per year (see Craig and Swinton [11] for a summary of testing observations). Of the players analysed, 100 were awarded professional contracts, 50 remained within the academy system and 362 were released or dropped out at the time of analysis. Within the participant group, a strong relative age bias has been previously identified in players awarded professional contracts (Q1 = 0.50; Q2 = 0.26; Q3 = 0.20; and Q4 = 0.05 ($\chi_{3}^{2}$= 40.9, p < 0.001)), potentially due to the relative age bias in the entire group that existed (Q1 = 0.37; Q2 = 0.28; Q3 = 0.22; and Q = 0.13 ($\chi_{3}^{2}$= 64, p < 0.001)) [11]. Q1 was defined as players born January to March, Q2 April to June, Q3 July to September and Q4 October to December as the section year operates an annual calendar categorisation with all players being born January – December being included in the same age grouping.

### Physiological testing

Participants were tested at the same location, on the same surface at the same time of day throughout the period investigated. An initial measure of stature to nearest 0.1 cm (SECA Height Measure, Hamburg, Germany) and body mass to 0.1 kg (SECA floor scale, Hamburg, Germany) was taken by an ISAK accredited anthropologist prior to a group standardised warm up. The greatest counter movement jump height (CMJ) of three repetitions from a self-selected counter movement depth was recorded to 0.1 cm (MuscleLab IR Jump MAT, Ergotest, Langonsund, Norway). Prior to the sprint protocol, participants completed 3x15m warm up runs (1x80%, 1x90%, 1x100% perceived maximum) followed by 3x20m maximal efforts with split times recorded to the nearest 0.01s at 5m, 10m and 20m (Speed Trap Timing, Brower Timing Systems, Utah, USA). The fastest 20m time and the associated 5m and 10m splits times were recorded for analysis. Sprints began 1m behind the 0m timing gate, were initiated with one foot self-selected in front of the other with a minimum of a 1:10 sprint: recovery period between the 3 trials. Following a ten minute recovery period, participants performed the YYIR1 test in groups that were set based on their previous YYIR1 results with test termination and final “score” following the recommended criteria. Comments related to appropriate test validity and reliability are provided in Craig and Swinton [11].

### Statistical analysis

Data were analysed using R statistical software [21]. A one-way analysis of variance (ANOVA) was performed to assess the impact of time and evolution of RAE changes across the seasons recorded. When the ANOVA indicated significant differences, post hoc comparisons were conducted using the Bonferroni correction to control for Type I error across multiple comparisons.

Effect sizes were calculated using eta squared (η²) to determine the magnitude of the differences observed. An η² of 0.01 was considered a small effect, 0.06 a medium effect, and 0.14 a large effect [22]. Pearson’s Chi squared tests were utilised across each season to investigate the impact of birth quarter on players ability to gain a professional contract. Relative age analysis was conducted for each age group (U10 to U17) using odds rations (OR) and 95% confidence intervals (95% CI) to calculate between quartile comparisons with each quartile being utilised as a referent group. A linear mixed model (LMM) was conducted for each physical testing variable using the lme4 package [23]. In this two-level hierarchical structure, players were treated as the nesting level (i.e., tests, players). Within this framework, the player variable was modelled as a random effect. Each physical test served as the dependent variable, while age group and quarter were fixed effects in the models. A general multilevel modelling strategy [24] was applied to each model, incorporating fixed and random effects incrementally from the simplest to the most complex models. The Akaike Information Criterion (AIC) [25] was utilised for model comparison at each step, with lower AIC values indicating a better model. Chi-square likelihood ratio tests were also used to compare models. Maximum likelihood (ML) estimation was applied for model comparison, and restricted maximum likelihood (REML) estimation was used to refit the final best model for each physical testing variable [24]. Marginal and conditional R^2^ values were reported for each significant LMM to indicate effect size [26,27]. The significance level was set at α = 0.05 for all statistical tests.

## Results

**Fig 1:**
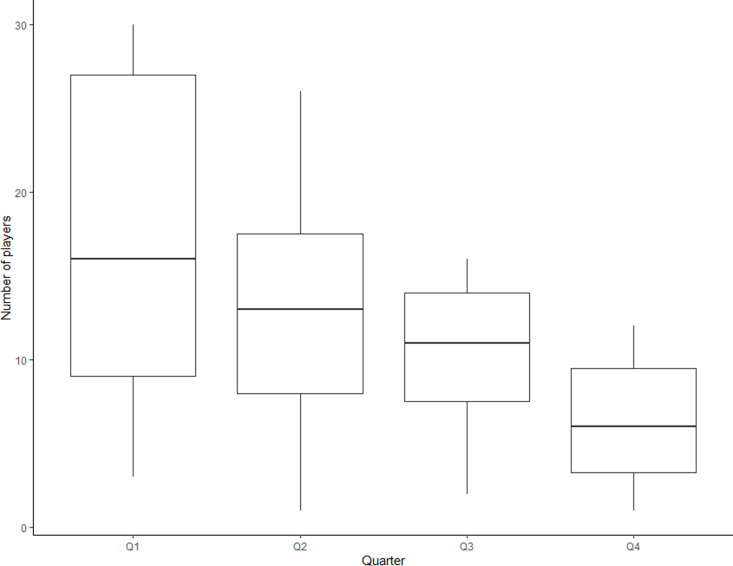
Distribution of relative age effect in entire sample.

**Fig 2:**
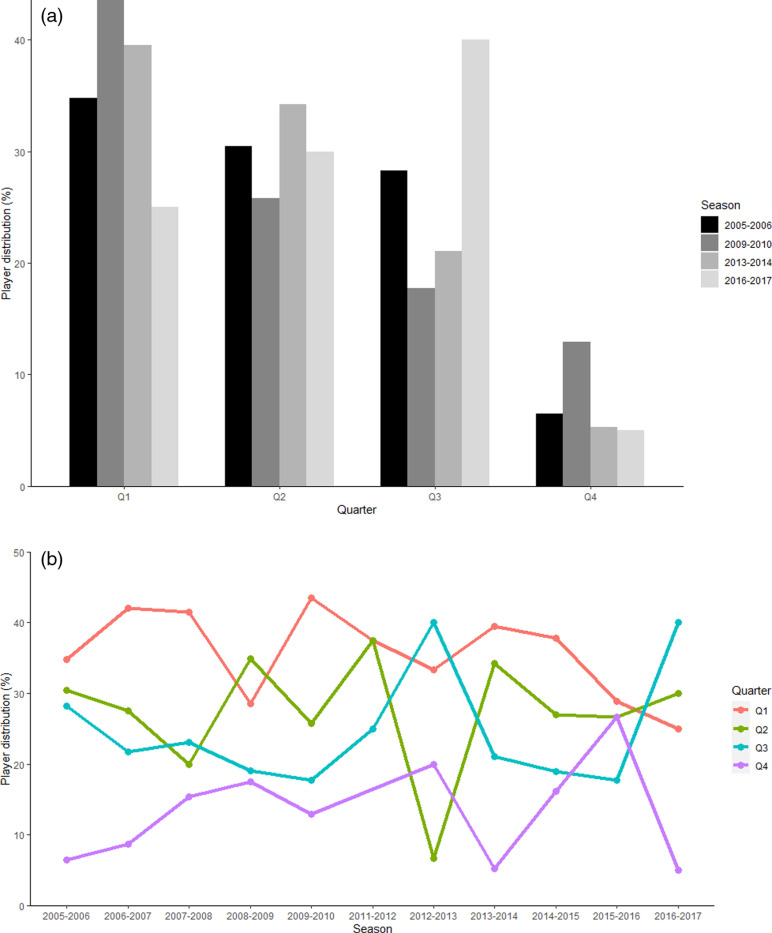
(A) The evolution of the RAE across the seasons investigated. (B) Annual Relative Age Effect Cohort.

In order to present a summary of the relative age effect in the entire academy being investigated, Fig 1 shows the distribution of the RAE across quarters for the whole sample. ANOVA and post hoc tests showed no significant effect of time in changes in RAE (*p* = 0.3217*)* with Fig 2A showing no obvious change in the annual cohorts. Although Fig 2B shows occasional cohort spikes across the time period, the pattern of high Q1 and low Q4 numbers remains throughout with no significant effect of time in changes in RAE. Whether assessed as entire sample (*p* = 0.2287) or annually (*p* = 0.1516), the Pearson’s Chi Squared tests showed that there was no significant effect of birth quarter on ability to be awarded a professional contract with their being no difference from the overall total player pool.

The birth quartile distributions and OR analysis for each age group is shown in Table 1. As expected, the sample shows a decreasing number of players at each age group as the squads progress from Q1 to Q4, which is apparent at all age group with the exception of U10s (Q3 (11.9%) and Q4 (14.3%) reversed) and U17s (Q2 (22.8%) and Q3 (25%) reversed). The range of players in each quarter was Q1 37–42.9% versus Q2 at 22.8–32.4%, Q3 at 11.9–26% and Q4 at 7.1–14.3%. With the exception of U10s (Q1vsQ3) OR analysis indicate a greater chance of selection within the academy when assessing Q1 vs Q4 players quarter comparisons (ranging 3.2–5.2 times more likely to be part of the academy). Q4 comparisons against Q1, Q2 and Q3 almost always show a significant difference regardless of the age group whereas Q1 vs Q2 only shows a significant difference at U17s indicating that the academy may be experiencing an under representation of Q4s rather than an over representation of Q1s.

**Table 1:** Birth date distribution of per quartile (Q) and age category (n(%)) by age group.

Age group		Birth distribution no (%)				Odds Ratios (95% CI)					
	N	Q1	Q2	Q3	Q4	Q1 vs Q4	Q2 vs Q4	Q3 vs Q4	Q1 vs Q2	Q1 vs Q3	Q2 vs Q3
10	42	18 (42.9)	13 (31.0)	5 (11.9)	6 (14.3)	3.0 (1.08-8.30)*	2.17 (0.75-6.24)	0.8 (0.42-2.94)	1.4 (0.60-3.18)	3.6 (1.22-10.59)*	2.6 (0.85-7.94)
11	170	68 (40.0)	49 (28.8)	32 (18.8)	21 (12.4)	3.2 (1.90-5.52)*	2.3 (1.34-4.06)*	1.5 (0.84-2.75)	1.4 (0.91-2.12)	2.1 (1.33-3.40)*	1.5 (0.93-2.51)
12	152	61 (40.1)	47 (30.9)	26 (17.1)	18 (11.8)	3.4 (1.91-6.00)*	2.6 (1.45-4.70)*	1.4 (0.76-2.74)	1.3 (0.83-2.02)	2.3 (1.41-3.91)*	1.8 (1.06-3.07)*
13	170	64 (37.6)	55 (32.4)	37 (21.8%	14 (8.2)	4.6 (2.47-8.47)*	3.9 (2.10-7.33)*	2.6 (1.38-5.07)*	1.2 (0.77-1.77)	1.7 (1.10-2.73)*	1.5 (0.93-2.37)
14	176	67 (38.1)	49 (27.8)	43 (24.4)	17 (9.7)	3.9 (2.22-6.98)*	2.9 (1.60-5.20)*	2.5 (1.39-4.60)*	1.4 (0.90-2.09)	1.6 (1.01-2.41)*	1.1 (0.72-1.80)
15	154	57 (37.0)	46 (29.9)	40 (26.0)	11 (7.1)	5.2 (2.62-10.26)*	4.2 (2.09-8.38)*	3.6 (1.80-7.35)*	1.2 (0.79-1.94)	1.4 (0.90-2.26)	1.2 (0.71-1.86)
17	136	56 (41.2)	31 (22.8)	34 (25.0)	15 (11.0)	3.7 (2.01-6.92)*	2.1 (1.07-4.00)*	2.3 (1.18-4.35)*	1.8 (1.10-2.98)*	1.6 (1.01-2.68)*	0.9 (0.53-1.57)
All groups	1000	391 (39.1)	290 (29.0)	217 (21.7)	102 (10.2)	3.8 (3.03-4.85)*	2.8 (2.23-3.62)*	2.1 (1.66-2.73)*	1.3 (1.13-1.61)*	1.8 (1.49-2.17)*	1.3 (1.10-1.63)*

Q1: Jan-Mar; Q2: Apr-Jun; Q3: Jul-Sep; Q4: Oct-Dec; OR: Odds Ratio Calculation; CI: 95% Confidence Interval. *significant difference (P < 0.05).

When controlling for age group multilevel modelling showed there were no significant differences across quarters in height, weight, BMI, 5m, 10m or 20m sprint and YYIR1 distance. There was however a statistically significant difference across quarters for CMJ (Table 2) with decreasing values shown in quarters throughout the year.

**Table 2:** Effect of age group and quarter on counter movement jump.

	CMJ			
Fixed effects	β (SE)	95% CI	t	p
Intercept	−0.62 (0.80)	−2.18, 0.94	−0.78	0.43
Age group	2.21 (0.06)	2.10, 2.32	39.39	<0.001
Q2	−0.12 (0.40)	−0.90, 0.67	−0.30	0.77
Q3	−1.00 (0.43)	−1.85, −0.15	−2.31	0.02
Q4	−1.44 (0.54)	−2.51, −0.38	−2.67	0.008
Randon effects	Estimate	SD		
Player	9.07	3.01		
Residuals	13.55	3.68		
R^2^_(m)_	0.48			
R^2^_(c)_	0.69			

## Discussion

The aim of this study was to establish any changes of the RAE previously evidenced within a Scottish soccer academy over a ten year period. The impact of the RAE on professional contracts awarded was also established and a further aim was to assess whether physical differences exist across each birth quarter. The main findings were that there was no impact of time with the RAE and the RAE did not further impact the already biased initial pool of players when awarding a professional contract. Odds ratio analysis indicated an underrepresentation of Q4 born players and there were no physical differences across quarters with the exception of CMJ which showed small differences. The lack of progress despite the increased knowledge of the RAE [17] during these periods is similar to what elite European clubs experience, but it is still disappointing.

The current analysis revealed no changes in the RAE over the ten year period reflecting the results observed in the first teams of Europe’s top leagues [10]. The finances associated with the top leagues and clubs in Europe are significantly higher than experienced in Scotland [28]. Indeed, in terms of UEFA Champions League distribution, the top 4 leagues have taken 61% of the revenue within the timeframe associated with the current study until 2016 with FC Barcelona averaging €246 million from UEFA Champions League group stage performance alone before considering sponsorship, ticketing, domestic and other income sources [28]. Although categorised as “elite” by the Scottish FA, the entire annual academy spend within the current group is estimated at £200-250k with spend allocated for “sports science support” ranging from £15-20k. The top leagues and clubs in Europe have the resources for interventions, technology and staffing to ensure efficiency in their talent development system. The FC Barcelona academy analysis evidenced a significant RAE at each age group of their academy and first team [8]. The odds ratio analysis evidenced the impact of this with the highest level being recorded that U10 Q1s were over 18 times more likely to be part of the FC Barcelona Academy than their Q4 born peers. The FC Barcelona analysis however only presents the odds ratio calculations with Q1, Q2 and Q3 versus Q4. The current Scottish study assesses the odds ratio between all quarter pairings. When assessing the odds ratio between Q3 and Q4, Q3 born players were 2.1–3.6 times more likely to be part of the academy from U13 onwards (P < 0.05). The RAE effect is often characterised as an over representation of players born earlier in the year [1] which is supported with by the annual patterns observed in the current analysis (Fig 2B). Regardless of the spikes shown in particular annual cohorts, the annual observations in Fig 2B also show that Q4 players are consistently lower across the time period. The authors of the current study propose that by changing the narrative as an under representation of late born players, this may impact the mindset and engage clubs to consider cost effective mechanisms to increase their efficiency and reduce dropouts of players with potential.

Collectively the literature suggests there are innovation opportunities for clubs and organisations to enhance the competitiveness and succession rates within their talent development pathways. Costly interventions that have been evidenced to be successful include the implementation of statistical modelling to remove the RAE from time performance sports [29,30] the creation of late maturing “future” squads in U16 and U17 youth international teams [10] and a manipulation of chronological banded categories [10]. Moving to 3 and 6 month age bands incurs significant costs not available to low income clubs and the perception that RAE interventions can be costly may be contributing to the prevalence of the RAE within low income environments. Low-cost initiatives include bib numbering aligned with age within trial and selection events [31,32], introducing an ability to play up or play down an age group [4] and introducing “selection quotas” [33].

Although evidence that supports selection quotas is limited [33], the authors propose that this could be utilised by teams in low income environments to ensure that players not fully developed are not removed on the basis of relative age. Keeping players that are not at the required level may create the perception that they are taking the place of a potential new recruit. Within the current group, 19.5% of players were awarded professional status into the club’s senior squads and within the English Premier League (EPL) academy system and feeder clubs, players have a 0.012% chance of being signed by a professional EPL club [34]. The risk that keeping players due to their relative age may decrease the risk of academy success for other players is thus small. When RAE is not considered by clubs who traditionally make subjective decisions about progression throughout the academy [35], these subjective decisions are subconsciously impacted by birth date and the perceived impact interferes with developmental opportunities such as progression and game time [12,31,35], a potential contributory factor as to why the RAE effect persists at high and low income soccer clubs. To alleviate this, we propose keeping the best 2–3 players of each quarter available to clubs and managing their development by playing them down an age category if required [4] to allow players to reach their full potential. An “underdog hypothesis” has been established that evidences players born later in the year may have more successful careers within professional sport [36], further supporting the position of our proposal. Where maturation onset rather than relative age creates developmental challenges, then exposure to bio banding opportunities can enhance the developmental opportunities [37] and clubs should ensure they have made a maturation analysis before their decision to release [38]. Decision makers at low income clubs cannot afford to miss out on potential first team players and should consider the lack of positive development in the RAE as an opportunity to increase efficiency.

Despite strong evidence of the RAE in academy soccer, there is evidence of younger (e.g., Q4) players still developing the appropriate performance levels for continued selection and progression towards professional status [13,15]. That this is particularly at the earliest stages of recruitment supports the observations of Hill [16] who observed a selection bias towards both RAE and early maturing players within another elite English academy and it is plausible that the Q4 players who match their older peers physically, do so as a result of the physical advantages of early maturation. Multilevel modelling across anthropometric and physical performance in the current study showed a difference in only CMJ between quarters mirroring similar work evidencing that early born players do not necessarily hold a physical advantage [13,15]. The differences in CMJ although statistically significant would likely have been physiologically trivial. The number of Q4 born players averaged at 8.2–14.3% of their respective age matched cohorts and our analysis suggests they would have been capable of competing physically with their older peers within the group. Although the current study has not analysed maturity status, it is likely that the younger players have survived drop out and deselection as early maturation provides the physical attributes to compete at the required level [14,15].

The main limitation within the current study is the absence of maturation information and control within the analysis. The current findings showing an absence of physical differences across quarters is likely due to the relatively younger players being advanced in their maturation status. The management of the sports science programme and testing within this academy was completed on a consultancy basis on a severely limited budget. Each additional assessment incurs a facility and time cost when clubs utilise a consultancy basis and even although the methods for maturity assessment can be completed non-invasively incurring mainly a time cost [39,40], there was no scope for additional funding for this assessment and monitoring within the time period monitored. Although the current study assesses relative age and physical performance in a single club, to the author’s knowledge this is the first longitudinal academy soccer study that does so in a low income environment and studies that have established changes in the RAE with time have not considered the associated physical progression. From a talent development perspective, it is not possible to simply repeat what has been completed elsewhere [18] and the understanding of where constraints such as the lack of resource in comparison with Europe’s top clubs does not impact efficiency and development is essential. Finally, although this study does not assess a single RAE intervention, across a ten year period it would be expected that multiple factors including continuous increased knowledge [10,17] would likely have contributed to the overall development of the academy.

## Conclusion

This study established whether there were changes in the RAE over a ten year period whilst also assessing physical differences across birth quarters within that timeframe. That there was no improvement in the RAE should be viewed as an opportunity that has not yet been seized. Cost effective interventions have been outlined that can be immediately implemented with potential to have a longitudinal impact, enhancing clubs’ recruitment and development success. Although the development of academy players is also beneficial for cash and resource rich clubs, it is more imperative for low income environments who rely on their academies for player and income generation. By addressing negative relative age, and where appropriate maturation onset challenges, we advise low income clubs to change the narrative and envisage an opportunity to enhance efficiency in an area that Europe’s elite clubs can spend to overcome.

## Supporting information

S1 Annon Test Data RAE minus ages (XLSX)
